# Evolution of laparoscopic liver surgery: 20-year experience of a Norwegian high-volume referral center

**DOI:** 10.1007/s00464-021-08570-3

**Published:** 2021-05-25

**Authors:** Davit L. Aghayan, Airazat M. Kazaryan, Åsmund Avdem Fretland, Bård Røsok, Leonid Barkhatov, Kristoffer Lassen, Bjørn Edwin

**Affiliations:** 1 The Intervention Centre, Oslo University Hospital - Rikshospitalet, 0027 Oslo, Norway; 2 Department of Surgery N1, Yerevan State Medical University After M. Heratsi, Yerevan, Armenia; 3 Institute of Clinical Medicine, Medical Faculty, University of Oslo, Oslo, Norway; 4 Department of Gastrointestinal Surgery, Østfold Hospital Trust, Grålum, Norway; 5 Department of Faculty Surgery, №2I.M. Sechenov First Moscow State Medical University, Moscow, Russia; 6 Department of HPB Surgery, Oslo University Hospital - Rikshospitalet, Oslo, Norway; 7 Department of Gastrointestinal Surgery, Haukeland University Hospital, Bergen, Norway

**Keywords:** Laparoscopy, Liver surgery, Liver resection, Laparoscopic liver resection

## Abstract

**Background:**

Laparoscopic liver surgery has evolved to become a standard surgical approach in many specialized centers worldwide. In this study we present the evolution of laparoscopic liver surgery at a single high-volume referral center since its introduction in 1998.

**Methods:**

Patients who underwent laparoscopic liver resection (LLR) between August 1998 and December 2018 at the Oslo University Hospital were analyzed. Perioperative outcomes were compared between three time periods: early (1998 to 2004), middle (2005 to 2012) and recent (2013–2018)**.**

**Results:**

Up to December 2020, 1533 LLRs have been performed. A total of 1232 procedures were examined (early period, *n* = 62; middle period, *n* = 367 and recent period, *n* = 803). Colorectal liver metastasis was the main indication for surgery (68%). The rates of conversion to laparotomy and hand-assisted laparoscopy were 3.2% and 1.4%. The median operative time and blood loss were 130 min [interquartile range (IQR), 85–190] and 220 ml (IQR, 50–600), respectively. The total postoperative complications rate was 20.3% and the 30-day mortality was 0.3%. The median postoperative stay was two (IQR, 2–4) days.

When comparing perioperative outcomes between the three time periods, shorter operation time (median, from 182 to 120 min, *p* < 0.001), less blood loss (median, from 550 to 200 ml, *p* = 0.023), decreased rate of conversions to laparotomy (from 8 to 3%) and shorter postoperative hospital stay (median, from 3 to 2 days, *p* < 0.001) was observed in the later periods, while the number of more complex liver resections had increased.

**Conclusion:**

During the last two decades, the indications, the number of patients and the complexity of laparoscopic liver procedures have expanded significantly. Initially being an experimental approach, laparoscopic liver surgery is now safely implemented across our unit and has become the method of choice for surgical treatment of most liver tumors.

Laparoscopic surgery has changed surgical practice over the last 30 years. The widespread interest also reached the hepato-pancreato-biliary (HPB) field, with the first laparoscopic liver resections (LLR) reported in early 1990-s [1, 2]. Later, case series, comparative studies, and multicenter reports demonstrated that LLR had the same advantages as reported in other surgical sub-specialties [3]. However, in addition to technical challenges, the spread of laparoscopic surgery for liver malignancies was delayed by concerns regarding resection margins, the risk of disease dissemination (implantation metastases), and difficulties in detecting small metastases.

Despite the initial skepticism, the number of LLRs has increased steadily, for both minor and major resections, as well as hepatectomies for living liver donation [4]. In a review of laparoscopic liver resections in 2009 [3], over 2800 procedures were reported and in another review in 2016 [5], the number of LLRs reached 9000.

To date, three consensus and guideline meetings on laparoscopic liver surgery have been held. At these meetings, leading experts have determined the optimal indications and conditions for performing LLR and provided recommendations on the further development and implementation of these procedures [4, 6, 7].

The first LLR in Norway was performed in 1998 followed by the first report in 2001 [8] including 11 procedures. Since then, the number of LLRs has increased exponentially and laparoscopic approach has become the treatment of choice for various malignant and benign liver tumors at our institution.

The aim of the current study was to analyze the evolution of LLR since its first introduction at Oslo University Hospital, Oslo, Norway.

## Materials and methods

### Patients

Oslo University Hospital is the only referral center for hepato-pancreato-biliary procedures for the South-East region of Norway, with a population of 3 million. In this study, we retrospectively reviewed our prospectively collected single-center database of laparoscopic liver resections over 20-year period. This study was approved by the local institutional review board and written consent from patients was not required due to the retrospective nature of the study.

**Fig. 1:**
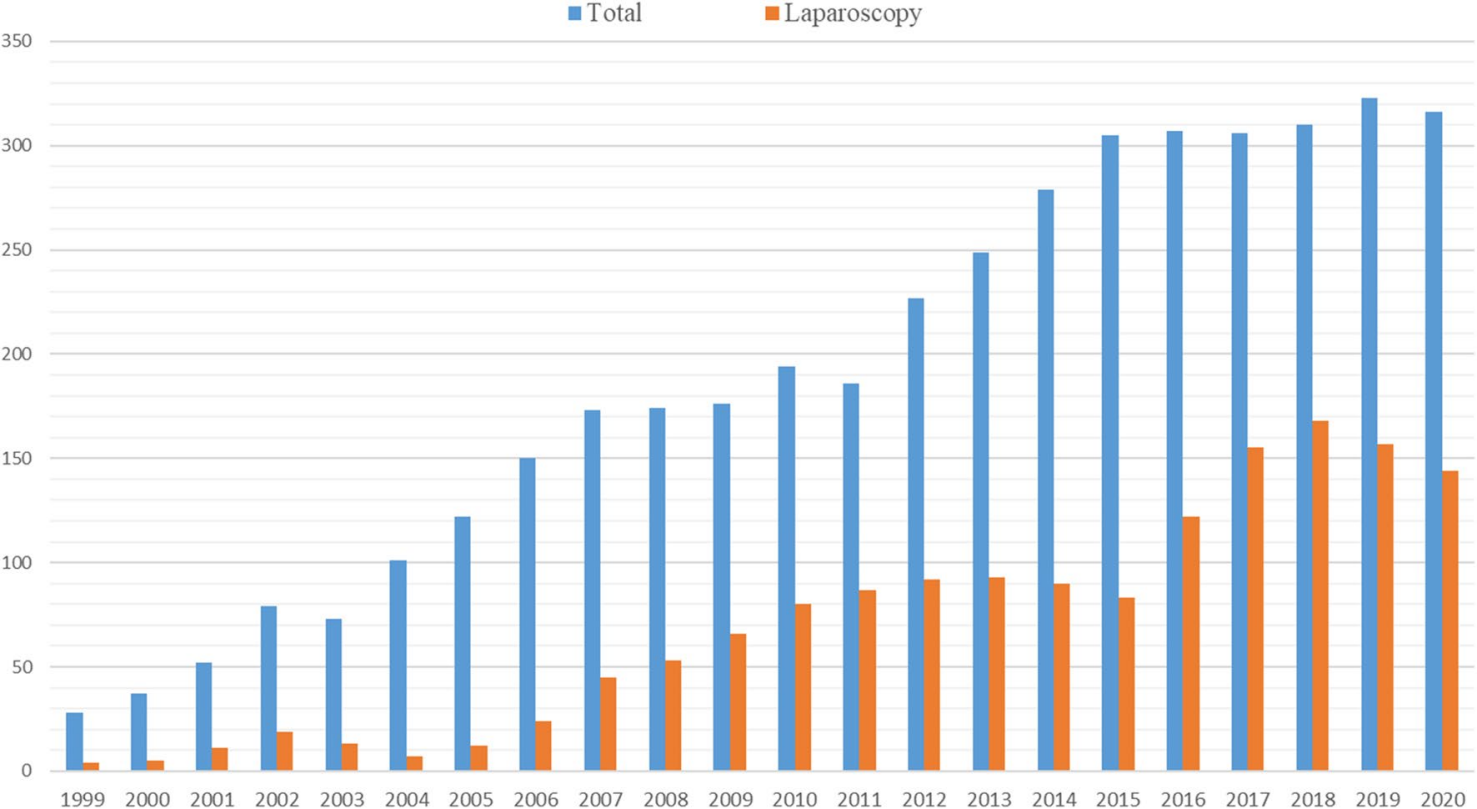
Annual number of liver resections from January 1999 to December 2020

Until December 2020, a total of 1533 laparoscopic liver resections have been performed (Fig. 1).

**Fig. 2:**
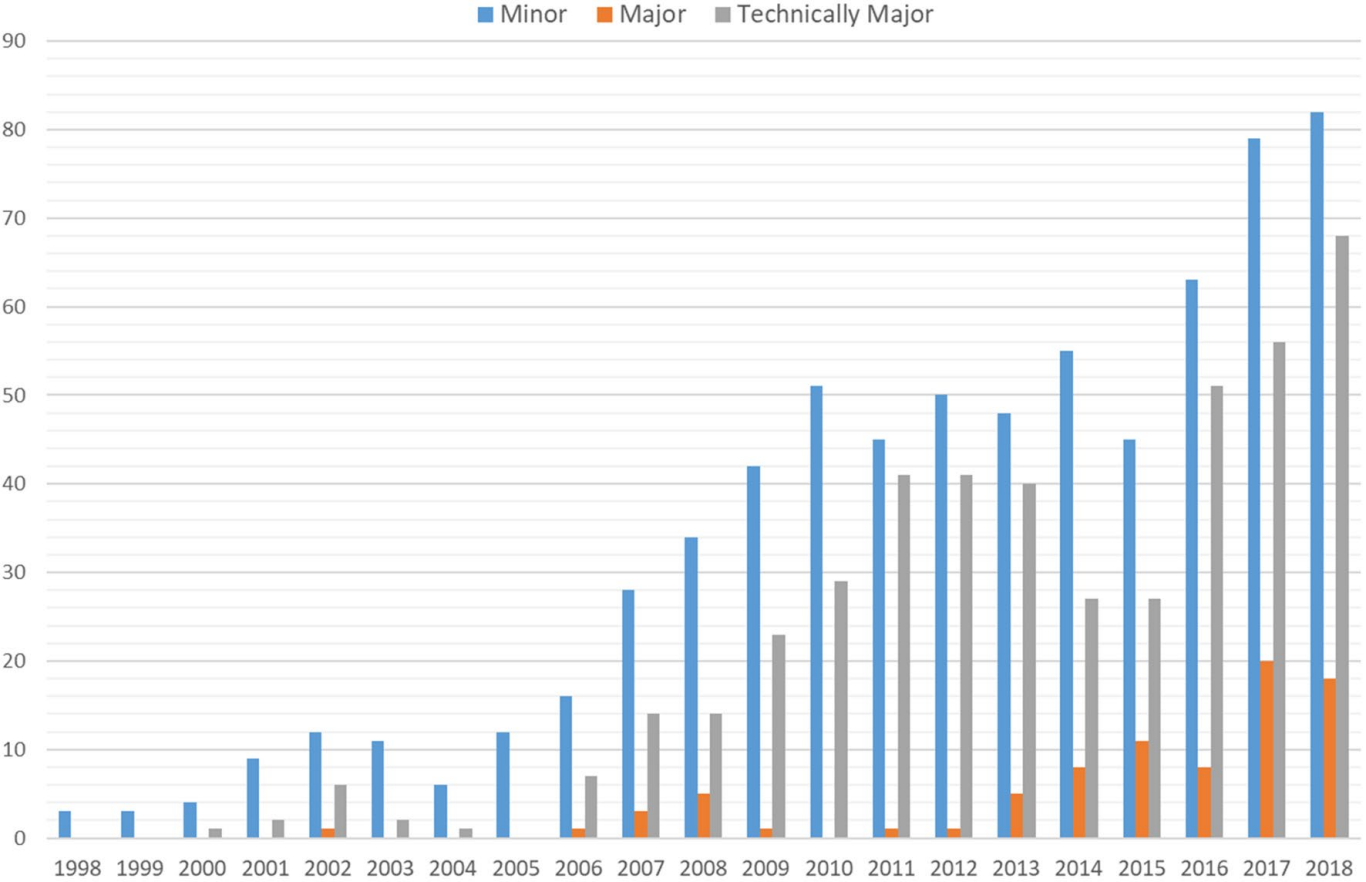
Extent of laparoscopic liver resections

Initially, laparoscopic liver resection was offered to patients planned for non-anatomic resections in the antero-lateral segments or for left lateral sectionectomy (minor resections). But with accumulation of surgical skills and development of laparoscopic technique, all types of liver resections have been considered for laparoscopy, including technically challenging (resection in the postero-superior segments) as well as larger anatomical resections (major hepatectomies involving more than 3 adjacent liver segments) (Fig. 2).

Perioperative management and surgical techniques have been described previously [9, 10]. In the current study, perioperative outcomes between three time periods (early, 1998 to 2004; middle, 2005 to 2012 and recent, 2013–2018) were compared**.** Data were collected from Electronic Health Records. Postoperative complications were registered as a dichotomous variable (yes/no) and the Accordion severity grading system of surgical complication was used to score postoperative morbidity by an independent medical specialist based on doctors and nurses records [11]. Survival rates of patients with colorectal liver metastases (CRLM), hepatocellular carcinoma (HCC) and intrahepatic cholangiocarcinoma (ICC) who primarily underwent laparoscopic liver resection is reported. Thus, the patients who previously had undergone liver resection were excluded from the survival analyses.

### Statistical analyses

**Fig. 3:**
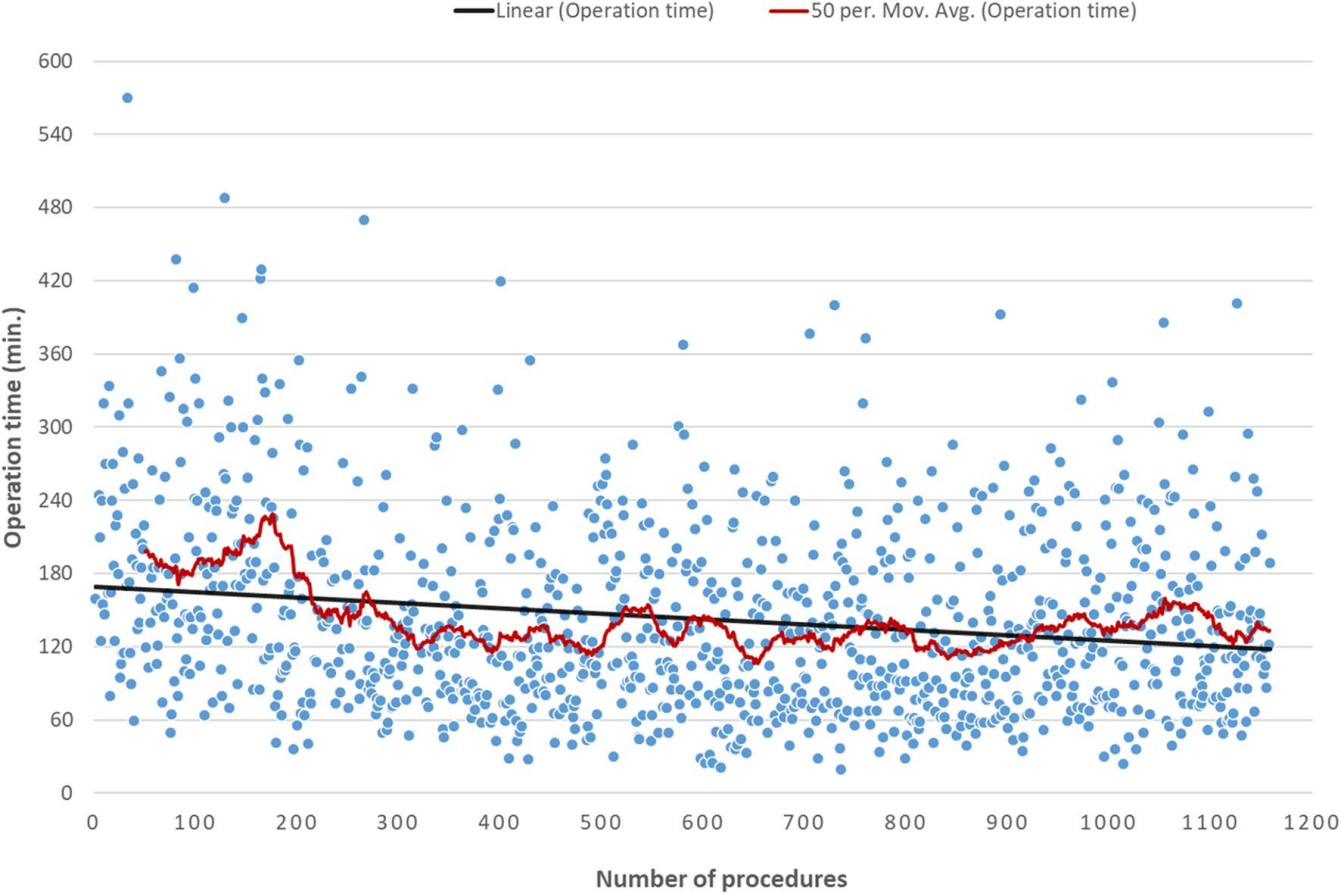
Dispersion graph of operative time: from 1998 to 2018 (cases with concomitant ablations were excluded)

Data are presented as median (IQR) and number (percentage). Case-specific operative time variation is presented in form of a dispersion graph with linear and moving average trendlines (Fig. 3). Categorical variables were compared using the Fisher’s exact test or the Chi-square test as appropriate. Continuous variables were compared using the Kruskal–Wallis test and One-Way ANOVA test for non-normally and normally distributed continuous data, respectively. Uni- and multivariate binary logistic regression analysis was performed to identify risk factors associated with postoperative complications. All variables associated with postoperative complications with *p* ≤ 0.2 in the univariate analysis were subsequently included into a multivariate regression model and *p*-values ≤ 0.05 were considered statistically significant.

Overall survival was estimated from the date of liver resection until death or censoring. Survival probabilities were calculated using the Kaplan–Meier method. Reverse Kaplan–Meier method was used to calculate median observation time. Time defined survivals are presented in percentage (± standard error). SPSS software (IBM Corp. Released 2013. IBM SPSS Statistics for Windows, version 25.0, Armonk, NY, USA: IBM corp.) was used for statistical analysis.

## Results

In total, 1232 laparoscopic liver resections in 1105 patients were analyzed. Colorectal liver metastases (CRLM) represented the main indication for surgery (68%). Other metastatic lesions were neuroendocrine metastases in 5%, melanoma metastases in 2%, other metastases in 4%. Hepatocellular carcinoma (HCC) in and intrahepatic cholangiocarcinoma (ICC) were the indications in 7% and 2% of the cases, respectively. Benign lesions were verified in 10% of cases (Table 1).

**Table 1:** Baseline characteristics and perioperative outcomes for the whole cohort

Variable	*N* = 1232
Gender, male/female	651/581
Age, y. median (IQR)	66 (56–73)
ASA, median (IQR)	2 (2–3)
BMI, median (IQR)	25.1 (22.7–28.4)
Indications	
CRLM	842 (68.3%)
Non-CRLM	132 (10.7%)
Benign lesions	122 (10%)
HCC	86 (6.9%)
Cholangiocarcinoma	22 (1.8%)
Other	28 (2.3%)
Type of liver resection	
Non-anatomic	967 (78.5%)
Anatomic	213 (17.3%)
Mixed	52 (4.2%)
Operation time, min, median (IQR)	130 (85–190)
Blood loss, ml, median (IQR)	200 (50–600)
Conversions	
Laparotomy, *n*	40 (3.2%)
Hand-assisted, *n*	21 (1.7%)
Total post op. complications, Grade ≥ I, *n*	250 (20.3%)
30 days mortality	4 (0.3%)
Post op. stay, median (IQR)	2 (2–4)
R1 (< 1 mm) resections, *n*^a^	208 (19%)
Involved resection margin^a^	103 (9.5%)

*IQR* interquartile range, *ASA* American Society of Anesthesiologists, *BMI* body mass index, *CRLM* colorectal liver metastasis, *HCC* hepatocellular cholangiocarcinoma

^a^Malignant liver tumors (n = 1082)

Anatomic resections were performed in 17%, non-anatomic parenchyma-sparing in 79%, and in the remaining 4% of cases, both anatomic and non-anatomic resections were performed in one procedure. Laparoscopic resections were combined with local ablations (either cryoablation or radiofrequency) in 72 cases. In 208 cases (17%), patients had previously undergone liver resection.

The median operative time and blood loss were 130 (IQR, 85–190) minutes and 200 (IQR, 50–600) ml, respectively. Rate of conversions to laparotomy was 3.2% and to hand-assisted laparoscopy 1.7%. Total postoperative complication rate (Accordion Grade ≥ I) was 20%. Postoperative 30-day mortality rate was 0.3%. The median postoperative stay was two (IQR, 2–4) days. For malignant tumors, R0 (≥ 1 mm) resection margin was achieved in 81% of the cases (Table 2).

**Table 2:** Indications and perioperative outcomes by time periods

Variable	Early Period 1998 to 2004 *n* = 62	Middle Period 2005 to 2011 *n* = 367	Recent Period 2012 to 2018 *n* = 803	*p* value
Indications, *n*				
CRLM	45 (72.6%)	251 (68.4%)	546 (68%)	0.759
Primary liver cancer^a^	1 (1.6%)	20 (5.4%)	87 (10.8%)	**0.002**
Benign lesions	10 (16%)	35 (9.5%)	77 (9.6%)	0.258
Age, media (IQR)	59 (54–70)	65 (56–73)	66 (56–73)	**0.026**
Male sex, *n*	32 (50%)	190 (51.7%)	429 (53.4%)	0.854
ASA score, median (IQR)	2 (2–3)	2 (2–3)	2 (2–3)	0.482
BMI, kg/m^2^, median (IQR)	24.4 (22.5–27.8)	25 (22.7–28.1)	25.2 (22.7–28.4)	0.998
Resection of multiple (> 1) lesions, n	11 (22.4%)	88 (26.6%)	252 (31.4%)	0.071
Operation time, min, median (IQR)	182 (138–245)	135 (90–200)	120 (81–180)	** < 0.001**
Blood loss, ml, median (IQR)	550 (200–1225)	250 (50–638)	200 (50–600)	**0.023**
Concomitant RF or Cryo-ablation, *n*	6 (9.7%)	18 (4.9%)	50 (6.2%)	0.284
Conversions, *n*				
Laparotomy	5 (8%)	7 (2%)	28 (3.4%)	**0.034**
Hand- Assisted	0 (0%)	7 (1.9%)	14 (1.7%)	0.639
Extent of resection				** < 0.001**
Minor	49 (79%)	228 (62%)	422 (52.6%)	
Left lateral sectionectomy	14	40	84	
Resection in AL segments	35	188	338	
Anatomically Major	1 (1.6%)	11 (3%)	71 (8.8%)	
Left hemihepatectomy	1	2	30	
Right hemihepatectomy	0	9	41	
Technically Major	12 (19.4%)	128 (35%)	310 (38.6%)	
Total morbidity, *n*	10 (16.1%)	70 (19%)	170 (21.2%)	0.581
Grade ≥ II, *n*	9 (14%)	53 (14.4%)	163 (20.3)	**0.038**
Severe (Grade ≥ III), *n*	4 (6.5%)	28 (7.6%)	95 (11.8%)	0.052
Readmission, *n*	3 (4.7%)	15 (4.1%)	64 (7.9%)	** < 0.001**
30-days mortality, *n*	0 (0%)	1 (0.3%)	3 (0.4%)	1.00
Post op. stay, days, median (IQR)	3 (3–4)	3 (2–5)	2 (2–3)	** < 0.001**
Median size of tumor, mm (IQR)	30 (24–40)	21 (15–35)	25 (15–40)	**0.010**
Large tumors (> 50 mm), *n*	9 (14.5%)	37 (10.1%)	129 (16.1%)	**0.024**
Median resection margin, mm (IQR)	5 (1–10)	3 (1–8)	3 (1–7)	**0.040**
Median weight of specimen, g (IQR)	92 (45–159)	51 (24–147)	60 (24–189)	0.153

*CRLM* colorectal liver metastasis, *IQR* interquartile range, *RF* radiofrequency, *AL* anterolateral

^a^Hepatocellular carcinoma and Intrahepatic Cholangiocarcinoma

When comparing perioperative outcomes between three time periods, the most recent period was associated with shorter operation time (median, from 182 to 120 min, *p* < 0.001), less blood loss (median, from 550 to 200 ml, *p* = 0.023), decreased rate of conversions to laparotomy (from 8 to 3%), shorter postoperative hospital stay (median, from 3 to 2 days, *p* < 0.001) and increased rate of severe (Grade ≥ 3) postoperative complications (from 6.5% to 11.8%, *p* = 0.052), while the number of more demanding liver resections had increased (Table 2, Fig. 2). It is worth to mention that the Pringle maneuver was mainly used at the end of the last period, especially in technically major resections. This may have led to a decreased blood loss in the recent period.

In multivariate analysis, patients age and extent of resection, particularly anatomical major resections, were independent risk factors for postoperative complication (Grade ≥ 2) (Table 3). Multivariate analysis for risk factors associated with severe postoperative complications (Grade ≥ 3) revealed that anatomical major resection was the only independent factor (Table 4). However, the most frequent severe complication among patients with anatomical major resection was fluid collection that required percutaneous drainage.

**Table 3:** Univariate and multivariate analysis of risk factors associated with Grade ≥ II complications

Variable	Univariate		Multivariate	
	Odds ratio (95% CI)	*p *value	Odds ratio (95% CI)	*p* value
Age (per year)	**1.02. (1.00 to 1.03)**	**0.013**	**1.02 (1.00 to 1.03)**	**0.016**
ASA score (1/2 vs 3/4)	**1.22 (0.91 to 1.65)**	**0.185**	1.09 (0.79 to 1.52)	0.588
Male sex	**1.2 (0.91 to 1.62)**	**0.196**	0.78 (0.57 to 1.07)	0.124
Malign tumor	1.25 (0.75 to 2.08)	0.399		
BMI, kg/m^2^	0.99 (0.96 to1.02)	0.446		
Multiple (> 1) lesions	**1.45 (1.06 to 1.98)**	**0.019**	1.35 (0.97 to 1.89)	0.079
Concomitant ablation	0.87 (0.46 to 1.64)	0.659		
Extent of resection				
Minor (ref.)				
Technically major	**1.23 (0.91 to 1.69)**	**0.183**	1.14 (0.81 to 1.59)	0.457
Anatomically major	**2.76 (1.67 to 4.56)**	** < 0.001**	1.96 (1.12 to 3.44)	**0.019**
Size of tumor, cm	**1.05 (1.00 to 1.11)**	**0.052**	1.04 (0.99 to 1.10)	0.145

*ASA* American Society of Anesthesiologists, *BMI* body mass index

**Table 4:** Univariate and multivariate analysis of risk factors associated with Grade ≥ III complications

Variable	Univariate		Multivariate	
	Odds ratio (95% CI)	*p *value	Odds ratio (95% CI)	*p* value
Age (per year)	**1.02. (1.01 to 1.03)**	**0.001**	1.01 (0.99 to 1.03)	0.164
ASA score (1/2 vs 3/4)	**1.43 (0.98 to 2.08)**	**0.060**	1.32 (0.87 to 1.99)	0.183
Male sex	0.97 (0.67 to 1.40)	0.867		
Malign tumor	1.49 (0.74 to 3.02)	0.265		
BMI, kg/m^2^	1.00 (0.96 to1.05)	0.875		
Multiple (> 1) lesions	1.27 (0.85 to 1.89)	0.236		
Concomitant ablation	**0.48 (0.17 to 1.34)**	**0.161**	0.58 (0.21 to 1.65)	0.308
Extent of resection				
Minor (ref.)				
Technically major	0.94 (0.62 to 1.41)	0.752	0.98 (0.64 to 1.51)	0.926
Anatomically major	**3.25 (1.86 to 5.66)**	** < 0.001**	**2.70 (1.46 to 5.00)**	**0.002**
Size of tumor, cm	**1.09 (1.03 to 1.16)**	**0.006**	1.07 (0.99 to 1.14)	0.060

*ASA* American Society of Anesthesiologists, *BMI* body mass index

**Fig. 4:**
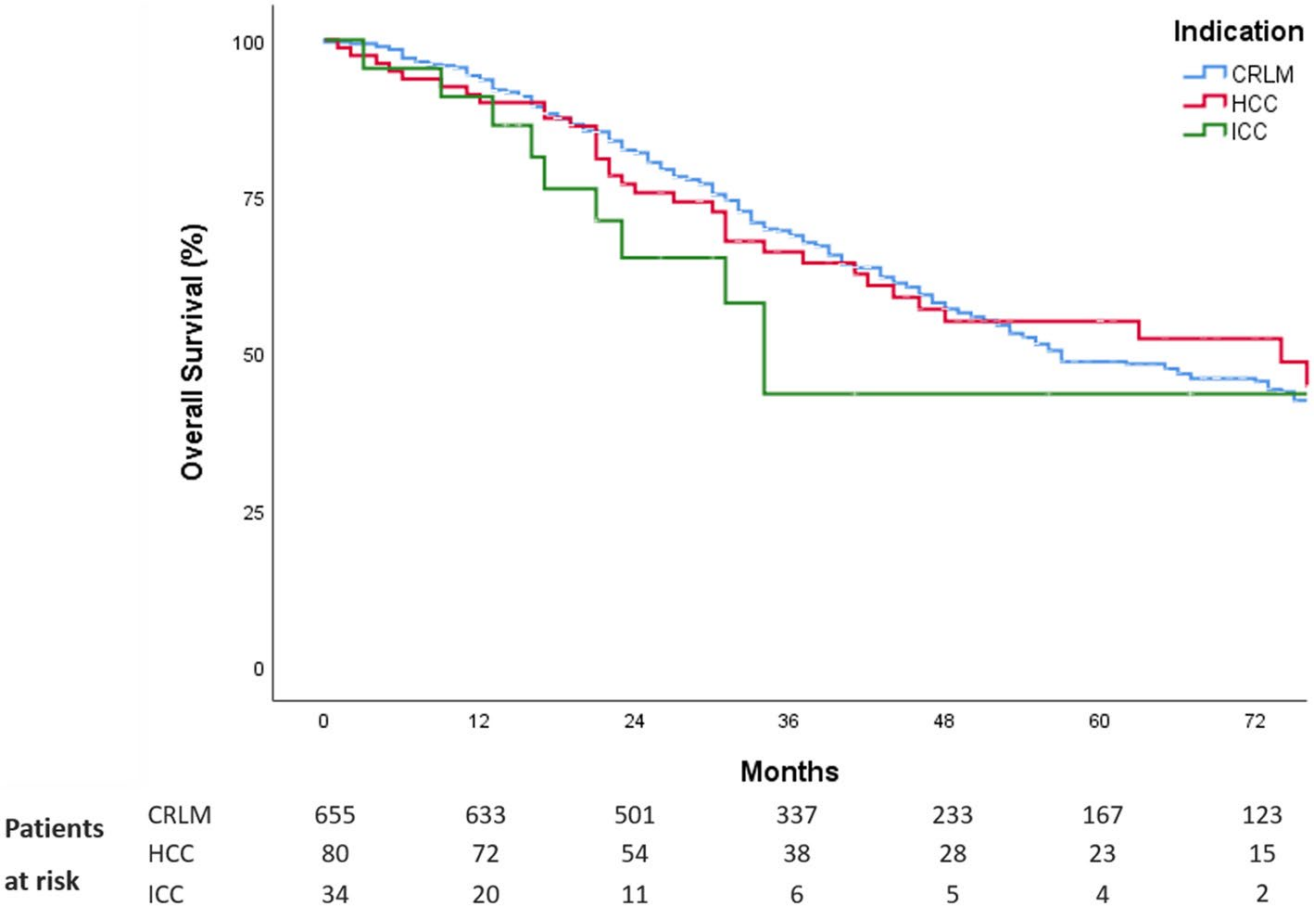
Kaplan–Meier survival curves for overall survival for patients with CRLM (*n* = 655), HCC (*n* = 80) and ICC (*n* = 34)

Median observation time for patients with CRLM, HCC and ICC were 43 [95% confidential interval (CI), 36–50], 60 (95%CI, 47–73) and 56 (95%CI, 20–91) months, respectively. The 5-year overall survival for patients with CRLM, HCC and ICC, who had LLR as the primary liver operation were 49% (± 2.6), 55% (± 6.2) and 44% (± 12.5), respectively (Fig. 4).

## Discussion

We here report over 20 years experience in laparoscopic liver surgery. Over time, an increase in numbers and complexity of laparoscopic procedures, and decreased operation time without an increase in the conversion rates, was found. Notably, the operation time continuously decreased which reflects that operative techniques are still in development. The longer operative time observed in the early period reflects the pioneering self-learning stage of laparoscopic liver surgery introduction in our institution (13). After a period of middle experience characterized by relatively narrow dispersion of operative time, a second, and less pronounced, increase of operative time dispersion was observed in the most recent period (Table 2; Fig. 3). Furthermore, an increase in postoperative complication rates of the Accordion Grade 2 or higher was observed in the later period. These may be explained both by an increasing number of surgeons operating, and by a significant increase of technically challenging resections. The new surgeons will be at different stages of their learning curve and technical expertise. Despite this, the operative time still decreases, while other perioperative outcomes are not compromised. This reflects a safe implementation of laparoscopic liver surgery as a routine treatment since its introduction in 1998.

In a multivariate analysis, variables that were significantly different in the later period (higher patient age and larger fraction of anatomical major resections), were independently associated with Grade ≥ 2 postoperative complications (Table 3).

The development and implementation of laparoscopic liver surgery was challenging and limited to expert centers. In the first consensus meeting held in Louisville in 2008, patients with solitary tumors smaller than 5 cm located in the antero-lateral liver segments were recommended for LLR [6]. In the Southampton consensus guidelines for laparoscopic liver surgery from 2017, the experts stated that tumor size and resections in the postero-superior segments (technically major resections) were risk factors for conversion and could be safely handled by surgeons with extensive experience in laparoscopic liver surgery. In our cohort, over the time periods, we found an increase of patients with multiple tumors, patients with large tumors, and patients that underwent major liver resection (Table 2).

As one can observe, the indications for LLR have changed significantly in our center over time, leading to more technically and medically demanding patients to be considered for laparoscopy. This can be associated with growing experience, significant improvement in surgical equipment and pre- and intraoperative imaging modalities, which improves preoperative resection planning and intraoperative navigation.

Interestingly, the median age of patients accepted for surgery has significantly increased in our series. More elderly patients who were previously denied an opportunity for a potentially curative liver resection are now offered surgery. This is in line with recent reports showing that LLR might be beneficial in elderly patients [12, 13].

In contrast to other surgical subspecialties, the development and implementation of laparoscopic technique in liver surgery has been relatively slow, likely, due to the demanding long learning curve [14, 15]. After the introduction of LLR in Norway in 1998, during an initial period of 8 years, there was solely one surgeon who performed or supervised all laparoscopic liver procedures at our hospital. Clear benefits of laparoscopic liver surgery were convincing and led to a growing need for establishment of training programs. Currently, seven surgeons independently perform laparoscopic liver resections, with different levels of expertise, The growing experience of our team, the results of our internal qualitative analyses and the worldwide interest in laparoscopic liver surgery inspired a prospective randomized controlled trial [8, 9, 16–18]. The OSLO-COMET trial demonstrated the advantages of LLR in patients with CRLM [10, 19–22].

Colorectal cancer liver metastases remain the most common indication for liver surgery in western countries, as in Norway [9, 23, 24]. The parenchyma-sparing strategy has become a first line surgical approach for these patients, carrying fewer complications, improving the possibility for repeat resections, and possible also improving survival, compared to formal hepatectomies [23, 25, 26]. Laparoscopic parenchyma-sparing surgery was established in the early phase when we started laparoscopic liver resections. However, despite the initial skepticism, the parenchyma-sparing approach has proved its importance, particularly in the multimodal treatment for the patients with CRLM [27, 28].

The current study has several shortcomings. First of all, this is a retrospective analysis with a possible information bias. The long study period is another limitation and differences in patient selection, surgical instruments, pre- and postoperative management of patients have been observed. The large difference in number of patients in the three study periods is another weakness and may lead to false-negative findings.

## Conclusion

During the last two decades, the indications, the number of patients and the complexity of laparoscopic liver procedures have expanded significantly. Initially being an experimental approach, laparoscopic liver surgery is now safely implemented across our unit and has become the method of choice for surgical treatment of most liver tumors.
